# Losing the Warning Signal: Drought Compromises the Cross-Talk of Signaling Molecules in *Quercus ilex* Exposed to Ozone

**DOI:** 10.3389/fpls.2017.01020

**Published:** 2017-06-15

**Authors:** Lorenzo Cotrozzi, Elisa Pellegrini, Lucia Guidi, Marco Landi, Giacomo Lorenzini, Rossano Massai, Damiano Remorini, Mariagrazia Tonelli, Alice Trivellini, Paolo Vernieri, Cristina Nali

**Affiliations:** ^1^Department of Agriculture, Food and Environment, University of PisaPisa, Italy; ^2^Institute of Life Sciences, Scuola Superiore Sant’AnnaPisa, Italy

**Keywords:** climate change, holm oak, mediterranean plant species, phytohormones, hypersensitive response, stress combination

## Abstract

Understanding the interactions between drought and acute ozone (O_3_) stress in terms of signaling molecules and cell death would improve the predictions of plant responses to climate change. The aim was to investigate whether drought stress influences the responses of plants to acute episodes of O_3_ exposure. In this study, the behavior of 84 Mediterranean evergreen *Quercus ilex* plants was evaluated in terms of cross-talk responses among signaling molecules. Half of the sample was subjected to drought (20% of the effective daily evapotranspiration, for 15 days) and was later exposed to an acute O_3_ exposure (200 nL L^-1^ for 5 h). First, our results indicate that in well-water conditions, O_3_ induced a signaling pathway specific to O_3_-sensitive behavior. Second, different trends and consequently different roles of phytohormones and signaling molecules (ethylene, ET; abscisic acid, ABA; salycilic acid, SA and jasmonic acid, JA) were observed in relation to water stress and O_3_. A spatial and functional correlation between these signaling molecules was observed in modulating O_3_-induced responses in well-watered plants. In contrast, in drought-stressed plants, these compounds were not involved either in O_3_-induced signaling mechanisms or in leaf senescence (a response observed in water-stressed plants before the O_3_-exposure). Third, these differences were ascribable to the fact that in drought conditions, most defense processes induced by O_3_ were compromised and/or altered. Our results highlight how *Q. ilex* plants suffering from water deprivation respond differently to an acute O_3_ episode compared to well-watered plants, and suggest new effect to be considered in plant responses to environmental changes. This poses the serious question as to whether or not multiple high-magnitude O_3_ events (as predicted) can change these cross-talk responses, thus opening it up possible further investigations.

## Introduction

Mediterranean plants are threatened by several abiotic stress factors [e.g., warming, drought, tropospheric ozone (O_3_), UV radiation, salinity] due to environmental changes characterized by new types of stress conditions and stress combinations, which are expected to be more extreme in the Mediterranean than in other areas worldwide (Matesanz and Valladares, 2014; Gray and Brady, 2016; Guidi et al., 2017). Today, drought is the major factor limiting plant performance, and revealing the mechanisms that enable plants to survive or acclimatize to such conditions is crucial (Claeys and Inzé, 2013). On the other hand, O_3_ is by far the most phytotoxic air pollutant with deleterious effects on growth and productivity (Vainonen and Kangasjärvi, 2015; Cotrozzi et al., 2016a; Yang et al., 2016). Both drought and O_3_ are co-occurring, increasing stressors in future climate change scenarios (Bates et al., 2008). Given that high-level O_3_ episodes and drought often occur together in Mediterranean areas, especially during the summer, their interaction needs to be understood (Iyer et al., 2013; Cotrozzi et al., 2016b). O_3_ enters the leaf through open stomata, then drought-triggered stomatal closure limits O_3_ uptake, thereby limiting foliar damage (Panek et al., 2002). However, other studies have highlighted that these factors have a synergistic effect, with increased O_3_ sensitivity observed in droughted plants (Alonso et al., 2014; Pollastrini et al., 2014). The response of plants to a combination of stresses is species-specific and depends on the intensity and duration of each stress factor (Ramegowda and Senthil-Kumar, 2015).

Plant exposure to acute O_3_ (high O_3_ concentration within a short period) commonly occurs during hot, dry Mediterranean summers (Matesanz and Valladares, 2014), which often results in a programmed cell death (PCD) response. This is a physiological process that selectively targets and eliminates unwanted cells in response to a variety of biotic and abiotic stimuli (Apel and Hirt, 2004). PCD resembles the hypersensitive response (HR) observed in several plant-pathogen interactions, which often precedes the acquisition of a systemic resistance by plants (Kangasjärvi et al., 1994; Rao et al., 2000; Pellegrini et al., 2013; Vainonen and Kangasjärvi, 2015; Pellegrini et al., 2016). O_3_ entering the leaves first induces a biphasic oxidative burst with a massive, rapid and transient increase in apoplastic reactive oxygen species (ROS), which is the main event leading to PCD activation (Langebartels et al., 2002) Similarly, an oxidative burst was usually observed in plants under drought (Smirnoff, 1993; Miller et al., 2010; Noctor et al., 2014) and the drought-triggered ROS production can elicit acclimatory events (Smirnoff, 1993). However, HR-like response has never been observed following drought stress. Therefore, the role of ROS in cell signaling and in regulating gene expression is a key aspect (Baxter et al., 2014), in particular in plant, subjected to abiotic stresses, including O_3_ and drought (Wilkinson and Davies, 2010).

The signaling pathways activated by O_3_ are integrated into a complex regulatory system involving ROS, plant hormones [e.g., ethylene (ET) and abscisic acid (ABA)], signaling molecules [e.g., salicylic acid (SA) and jasmonic acid (JA)], and secondary messengers (e.g., Ca^2+^). Signaling and cell death in O_3_-exposed plants have been reviewed by several authors (e.g., Rao et al., 2000; Rao and Davis, 2001; Kangasjärvi et al., 2005; Tamaoki, 2008; Vainonen and Kangasjärvi, 2015; Carmody et al., 2016; Pellegrini et al., 2016). Different plants use many hydraulic and chemical signals to tune their sensing of water deficit (Wilkinson and Davies, 2010). Thus, the interactions between drought and acute O_3_ stress in terms of signaling molecules and cell death need to be studied in depth in order to improve predictions of plant acclimation/adaptation strategies to climate change (Carmody et al., 2016). Signaling in acute O_3_ exposure has mainly been studied in the test plant *Arabidopsis thaliana*, however, few works have evaluated these mechanisms in tree species [e.g., on hybrid poplar, by Kock et al. (2000)].

To the best of our knowledge, no studies have assessed signaling molecules and cell death in Mediterranean tree species exposed to O_3_. O_3_ can also be used as a non-invasive tool to mimic signaling pathways triggered by active apoplastic ROS formation induced by pathogens (Vainonen and Kangasjärvi, 2015), also enabling conclusions to be drawn on drought-biotic stress interactions. The responses of Mediterranean species to the interaction of drought and O_3_ have yet to be extensively investigated as shown by the scarce information available in the literature (Kurz et al., 1998; Vitale et al., 2008; Calderòn Guerrero et al., 2013; Alonso et al., 2014; Cotrozzi et al., 2016b), especially in relation to acute exposure to the pollutant.

Holm oak (*Quercus ilex* L.) is probably the most widely studied Mediterranean evergreen tree species which has been defined as ‘drought-avoidant’ and ‘water saver’ with regard to its ecophysiological behavior (Bussotti et al., 2002), although adverse impacts of drought have also been reported in this species (e.g., Gimeno et al., 2008; Cotrozzi et al., 2016b). This species has also been referred to as the most tolerant to O_3_ stress among several other *Quercus* species (Calatayud et al., 2011). In a previous study carried out by this research group (Cotrozzi et al., 2016b), *Q. ilex* subjected to drought (30% of the effective daily evapotranspiration) and/or chronic O_3_ (80 nL L^-1^, 5 h d^-1^, for 77 consecutive days) showed that the major determinant was the water deficit; however, oxidative stress (revealed by a significant build-up of MDA by-products) occurred only when drought was applied with O_3_ (Cotrozzi et al., 2016b).

In the present study, we evaluated the behavior of *Q. ilex* saplings, subjected or not to drought, and later exposed to acute O_3_ exposure by characterizing different components of O_3_ stress signaling. Our aim was to answer the following questions: (i) can acute O_3_ exposure initiate an HR? (ii) What role do phytohormones and signaling molecules play in the perception and transduction of drought and/or O_3_ stress? (iii) Do drought conditions compromise/alter the signaling responses to acute O_3_ exposure?

## Materials and Methods

### Plant Material and Experimental Design

Three-year old *Q. ilex* saplings grown under field conditions were potted in 6.5-L pots with growing medium containing a mixture of standard soil Einhetserde Topfsubstrat ED 63 (Sinntal-Altengronau, Germany) and sand (3.5:1, in volume), according to Cotrozzi et al. (2016b). Two weeks before the beginning of the O_3_ treatment, 42 plants (WS) received 20% of the effective daily evapotranspiration (calculated by the average 24-h weight loss of five well-watered plants), whereas another 42 plants (WW) were kept at field water capacity. The two groups of plants were then subdivided into four sets (WW-O_3_, WS-O_3_, WW+O_3_, WS+O_3_; 21 plants per set) and transferred into four controlled fumigation facilities (temperature 23 ± 1°C, relative humidity 85 ± 5% and photon flux density of 530 μmol photons m^-2^ s^-1^ at plant height provided by incandescent lamps with L/D 14:10 photoperiod; lights were switched on from 7:00 to 21:00 to simulate environmental light conditions).

WW-O_3_ and WS-O_3_ plants were randomly distributed into two chambers, whereas WW+O_3_ and WS+O_3_ plants were randomly distributed in the other two chambers. After one week of acclimation, WW+O_3_ and WS+O_3_ plants were exposed to an acute O_3_ stress (200 nL L^-1^, 5 h day^-1^, in the form of a square wave between the 2nd and the 7th h of the light period). On the other hand, WW-O_3_, WS-O_3_ plants were maintained under charcoal-filtered air, in which the O_3_ concentration was less than 5 nL L^-1^. During the O_3_-exposure, environmental factors were maintained as reported above.

The O_3_ exposure was performed according to Lorenzini et al. (1994) with minor modifications to avoid pseudo-replications. At the end of the drought exposure, plant water status was evaluated. Photosynthetic parameters were measured at 0, 5, 24 and 48 h from the beginning of the O_3_ exposure (FBE, From the Beginning of Exposure). Five fully expanded mature leaves per plant per treatment were taken at 0, 1, 2, 5, 8, and 24 h FBE, stored at –20°C and subsequently used for chemical analyses, with the exception of ET determination, which was performed immediately. At the same measuring times, staining, and microscopic assays were also performed on fresh material.

### Water Status of Plants

Pre-dawn leaf water potential (PDΨ_W_) was measured on three plants per treatment (one fully expanded mature leaf per plant) with a pressure chamber (PMS model 600, PMS Instrument Company, Albany, OR, USA). On the very same plants, relative water content (RWC) was calculated (one fully expanded mature leaf per plant) as: RWC (%) = (FW-DW)/(TW-DW) × 100, where FW is the fresh weight, TW is the turgid weight after rehydrating samples for 24 h, and DW is the dry weight after oven-drying samples at 85°C for 24 h.

### Gas Exchange and Chlorophyll *a* Fluorescence Measurements

Gas exchange and chlorophyll *a* fluorescence measurements were determined between 10:00 and 13:00 (solar time) on one fully expanded mature leaf per plant, on three plants per treatment. CO_2_ assimilation rate (A), stomatal conductance to water vapor (*g*_s_) and intercellular CO_2_ concentration (*C*_i_) in light-saturated conditions and ambient CO_2_ concentration were measured using an Infrared Gas Analyzer (LI-COR Inc., Lincoln, NE, United States) as reported by Cotrozzi et al. (2016b). Modulated chlorophyll *a* fluorescence of photosystem II (PSII) was measured with a PAM-2000 fluorometer (Walz, Effeltrich, Germany) on the same leaves used for the gas exchange after 40 min of dark adaptation using a dark leaf clip provided by the producer. The maximal PSII photochemical efficiency [*F*_v_/*F*_m_ = (*F*_m_–*F*_0_)/*F*_m_] and the photochemical efficiency in light conditions [Φ_PSII_ = (*F*_m_’–*F*_s_)/*F*_m_’)] were calculated (Genty et al., 1989). Maximal fluorescence, *F*_m_, when all PSII reaction centers were closed, was determined by applying a saturating light pulse (0.8 s) at 8,000 μmol m^-2^ s^-1^ in dark-adapted leaves. Fluorescence was induced with actinic light (about 480 μmol m^-2^ s^-1^), superimposed with 800 ms saturating pulses (10,000 μmol m^-2^ s^-1^) at 20 s intervals to determine maximal fluorescence in the light-adapted state (*F’*_m_). Minimal fluorescence in the light-adapted state (*F’*_0_) was determined immediately after turning off the actinic source in the presence of a far-red (>710 nm) background for 10 s to ensure maximal oxidation of PSII electron acceptors. The saturation pulse method was used to analyze the quenching components, as described by Schreiber et al. (1986).

### Staining and Microscopic Assays

For the detection of dead cells, Evan’s blue staining was used according to Tonelli et al. (2015). Leaf material was boiled for 1 min in a mixture of phenol, lactic acid, glycerol and distilled water (1:1:1:1, in vol.) containing 20 mg mL^-1^ Evan’s blue and, after clarification with an aqueous chloral hydrate solution, examined under a light microscope (DM 4000 B, Leica, Wetzlar, Germany). To detect H_2_O_2_ accumulation, fresh leaf samples were stained with 3,30-diaminobenzidine (DAB) following Tonelli et al. (2015). Leaf parts were incubated for at least 8 h in a DAB solution (1 mg mL^-1^) in HCl adjusted to pH 5.6. The samples were then soaked in 70% (v/v) boiling ethanol and clarified overnight in a solution of 2.5 g L^-1^ aqueous chloral hydrate solution. The cellular H_2_O_2_ accumulation was visualized under a light microscope as a reddish-brown precipitation.

### ROS determination

H_2_O_2_ production was measured using the Amplex Red Hydrogen Peroxide/Peroxidase Assay Kit (Molecular Probes, Invitrogen, Carlsbad, CA, United States), according to Pellegrini et al. (2013). Spectrofluorimetric determinations were performed with a fluorescence/absorbance microplate reader (Victor3 1420 Multilabel Counter Perkin Elmer, Waltham, MA, United States) at 530 and 590 nm (excitation and emission resorufin fluorescence, respectively). $O_{2}^{–}$ concentration was measured according to Tonelli et al. (2015), after extraction with a Tris-HCl buffer (50 mM, pH 7.5), with a spectrophotometer (6505 UV-Vis, Jenway, United Kingdom) at 470 nm, and using a buffer solution as a blank.

### Phytohormone and Signaling Molecule Bioassays

Two minutes after excision, ET production was measured by enclosing six intact leaves (cut a few millimeters below the petiole by a scalpel) in air-tight glass containers (80 mL). Gas samples (2 mL) were taken from the headspace of containers after 1 h incubation at room temperature. Separations were performed with a gas chromatograph (HP5890, Hewlett-Packard, Ramsey, MN, United States) equipped with a stainless steel column (150 × 0.4 cm i.d. packed with Hysep T) and a flame ionization detector. Analytical conditions were as follows: injector and transfer line temperature at 70 and 350°C, respectively, and carrier gas nitrogen at 30 mL min^-1^ (Pellegrini et al., 2013). SA was determined according to Vitti et al. (2016) with some minor modifications. High performance liquid chromatography (HPLC) separations were performed with a liquid chromatograph (Dionex, Sunnyvale, CA, United States) equipped with a reverse-phase Dionex column (Acclaim 120, C18 5 μm particle size, 4.6 mm i.d. × 150 mm length) and RF 2000 Fluorescence Detector. Analytical conditions were as follows: excitation and emission at 305 and 407 nm, respectively, mobile phase containing 0.2 M sodium acetate buffer, pH 5.5 (90%) and methanol (10%), and the flow-rate at 0.8 mL min^-1^. JA was determined according to Pellegrini et al. (2013). HPLC separations were performed with the Dionex column described above and a UVD 170U UV/VIS detector. Analytical conditions were as follows: absorbance at 210 nm, mobile phase containing 0.2% (v/v) acidified water, and the flow-rate at 1 mL min^-1^. ABA was measured after extraction in distilled water (water:tissue ratio, 10:1) overnight at 4°C. The indirect ELISA determinations, based on the use of DBPA1 monoclonal antibody, raised against S(+)-ABA, as described by Trivellini et al. (2011), were performed at 415 nm with a microplate reader (MDL 680, Perkin-Elmer, Waltham, MA, United States).

### Proline Content

Proline content was measured as reported in Cotrozzi et al. (2016b), after extraction with sulfosalicylic acid (3%, v/v). Spectrophotometric determinations were performed at 520 nm, using toluene as a blank.

### Statistical Analysis

Three repeated experiments were set up following a randomized design and the experimental plot consisted of one plant per container. Ecophysiological and biochemical measurements were carried out on three replicates for each treatment. The normality of data was preliminary tested by the Shapiro–Wilk *W* test. The effects of drought exposure vs. well-watering were analyzed by the Student’s *t*-test. The effects of O_3_ on ecophysiological parameters were tested using one-way repeated measures ANOVA with treatment (WW+O_3_, WS+O_3_) as the variability factor. The effects of O_3_ on biochemical parameters were evaluated by two-way ANOVA with treatment and time as variability factors. For both ecophysiological and biochemical analyses, Fisher’s LSD was used as the post-test, with a significance level of *P* ≤ 0.05. Since data obtained by control plants maintained in filtered air (WW-O_3_ and WS-O_3_) did not show significant differences during the time course (*data not shown*), a comparison among means was carried out using as WW+O_3_ and WS+O_3_ plants controls before beginning the fumigation. Analyses were performed by NCSS 2000 Statistical Analysis System Software (Kaysville, UT, United States).

## Results

### Effects of Drought Stress

After 15 days of drought, plants did not show visible foliar injury. Physiological responses are reported in **Table 1**. In WS plants, PDΨ_W_ decreased significantly to -4.0 MPa at the end of water deprivation compared to WW controls (–0.5 MPa). However, no changes in RWC were recorded in WS leaves. The net carbon gain was reduced by about 73% in response to drought, which was a larger effect compared with the reduction of *g*_s_ (–50%). Values of *C*_i_ increased in WS leaves (+7%). Chlorophyll fluorescence measurements revealed a reduction in Φ_PSII_ (–39%) and qP (–18%) in WS compared to WW leaves, but no changes in the *F*_v_/*F*_m_ ratio. An increase of qNP (+30%) was found after drought stress.

**Table 1:** Water status and ecophysiological parameters in *Quercus ilex* plants well-watered (WW) or water stressed (20% of the effective evapotranspiration daily for 15 days, WS).

		WW	WS	*P*
PDΨ_W_	(–MPa)	0.5 ± 0.06	4.0 ± 0.70	^∗∗^
RWC	(%)	86 ± 7.4	82 ± 1.7	ns
A	(μmol CO_2_ m^-2^ s^-1^)	7.4 ± 0.23	2.0 ± 0.13	^∗∗∗^
g_s_	(mol H_2_O m^-2^ s^-1^)	0.16 ± 0.001	0.08 ± 0.008	^∗∗∗^
C_i_	(μmol CO_2_ mol^-1^)	284 ± 2.1	304 ± 8.1	^∗^
F_v_/F_m_		0.83 ± 0.003	0.84 ± 0.004	ns
Φ_PSII_		0.36 ± 0.008	0.22 ± 0.045	^∗∗^
qP		0.60 ± 0.008	0.49 ± 0.034	^∗∗^
qNP		0.64 ± 0.027	0.83 ± 0.043	^∗∗^

*Data are shown as mean ± standard deviation. Asterisks show the significance of Student’s *t*-test: ^∗∗∗^*P* ≤ 0.001, ^∗∗^*P* ≤ 0.01, ^∗^*P* ≤ 0.05, ns *P* > 0.05. Φ_*PSII*_, photochemical efficiency in light conditions; A, CO_*2*_ assimilation rate; Ci, intercellular CO_*2*_ concentration; F_*v*_/F_*m*_, potential PSII photochemical efficiency; g_*s*_, stomatal conductance to water vapor; PDΨ_*W*_, pre-dawn leaf water potential; qP, photochemical quenching coefficient; qNP, non-photochemical quenching coefficient; RWC, relative water content.*

The biochemical responses at the end of drought exposure are summarized in **Table 2**. In comparison to the controls, H_2_O_2_ levels did not change in WS leaves, while accumulation of $O_{2}^{–}$ was 1.6-fold higher under drought. A strong increase in Pro content (+39%) was observed in WS leaves. The endogenous concentration of ABA and SA measured in WS leaves decreased significantly at the end of the experimental period (–33% and –39%, respectively). However, the JA and ET amounts accumulated by WS leaves increased significantly (about 7-fold and +66%, respectively).

**Table 2:** Biochemical parameters in *Quercus ilex* plants WW or water stressed (20% of the effective evapotranspiration daily for 15 days, WS).

		WW	WS	*P*
H_2_O_2_	(μmol g^-1^ DW)	0.18 ± 0.011	0.17 ± 0.004	ns
$O_{2}^{–}$	(nmol min^-1^ g^-1^ DW)	24.0 ± 0.20	38.7 ± 1.47	^∗∗∗^
ET	(pl g^-1^ FW h^-1^)	136 ± 15.0	226 ± 10.8	^∗∗^
SA	(nmol g^-1^ DW)	7.1 ± 1.04	4.3 ± 0.08	^∗∗^
JA	(μmol g^-1^ DW)	3.5 ± 0.08	24.0 ± 0.35	^∗∗∗^
ABA	(nmol g^-1^ DW)	4.2 ± 0.03	2.8 ± 0.28	^∗∗∗^
Pro	(mmol g^-1^ DW)	0.23 ± 0.001	0.32 ± 0.010	^∗∗∗^

*Data are shown as mean ± standard deviation. Asterisks show the significance of Student *t*-test: ^∗∗∗^*P* ≤ 0.001, ^∗∗^*P* ≤ 0.01, ns *P* > 0.05. ABA, abscisic acid; DW, dry weight; ET, ethylene; FW, fresh weight; H_*2*_O_*2*_, hydrogen peroxide; JA, jasmonic acid; O_*2*_^-^, superoxide anion; Pro, proline; SA, salicylic acid.*

### Influence of Drought Stress on the Response to Acute O_3_ Exposure

#### Macroscopic and Microscopic Ozone-Induced Symptoms

**FIGURE 1:**
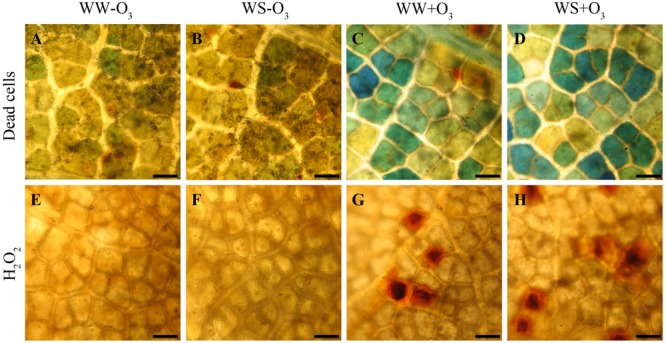
Localization of dead cells visualized with Evans blue staining **(A–D)** and of hydrogen peroxide (H_2_O_2_) visualized the 3,3′-diaminobenzidine (DAB) uptake method **(E-H)** in *Quercus ilex* leaves (i) well-watered (WW) and exposed to charcoal filtered air (WW-O_3_); (ii) water stressed (20% of effective evapotranspiration daily for 15 days) and exposed to charcoal filtered air (WS-O_3_); (iii) well-watered and exposed to acute ozone (200 nL L^-1^ for 5 h) (WW+O_3_); (iv) water stressed and O_3_ fumigated (WS+O_3_). The assays were performed 96 h FBE. Bars 50 μm.

At the end of the O_3_ treatment (alone and in combination with drought), leaves appeared macroscopically symptomless. However, O_3_-injuries were already detectable at the microscopic level after 5 h FBE, as confirmed by the appearance of dead cells observed in WW+O_3_ and WS+O_3_ (**Figures 1A–D**). Histological staining showed local accumulation of H_2_O_2_ evidenced by reddish-brown areas in O_3_-treated material (**Figures 1G,H**) (regardless of drought stress; **Figures 1E,F**).

#### Ozone-Induced Physiological Responses

**FIGURE 2:**
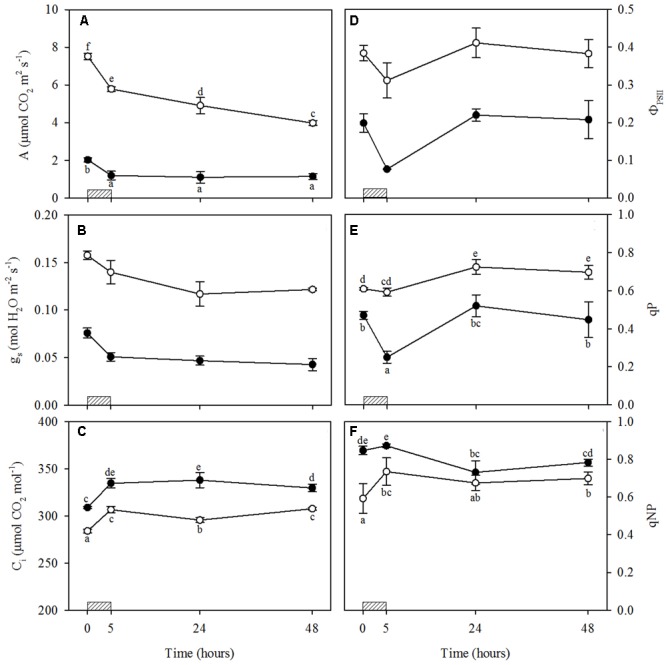
Time course of leaf gas exchange and chlorophyll fluorescence parameters in *Quercus ilex* plants well-watered (open circle) or water stressed (20% of the effective evapotranspiration daily for 15 days, closed circle) and exposed to acute ozone (200 nL L^-1^ for 5 h). Data are shown as mean ± standard deviation. The measurements were carried out 0, 5, 24 and 48 h from the beginning of exposure. According to the one-way repeated measures ANOVA with treatment as variability factor, different letters indicate significant differences (*P* = 0.05). The absence of letters in B and D indicates not significant interaction between variability factors (see Supplementary Table S1). **(A)** CO_2_ assimilation rate (A); **(B)** stomatal conductance to water vapor (g_s_); **(C)** intercellular CO_2_ concentration (C_i_); **(D)** photochemical efficiency in light conditions (Φ_PSII_); **(E)** photochemical quenching coefficient (qP); **(F)** non-photochemical quenching coefficient (qNP). The thick bottom line indicates the time (5 h) of ozone exposure.

The photosynthetic rate in light saturation conditions decreased strongly following O_3_ exposure in both WW+O_3_ and WS+O_3_ plants, and especially under drought (–23 and –41% in WW+O_3_ and WS+O_3_ plants, respectively) (**Figure 2A**). However, A values continued to decrease only in WW+O_3_ leaves, also after the end of fumigation, reaching values of about 4 μmol CO_2_ m^-2^s^-1^ at 48 h FBE with a reduction of about 50% compared to the values determined before O_3_ exposure (**Figure 2A**). WS+O_3_ plants showed low levels of A already before the beginning of O_3_ exposure (–73% in comparison with WW plants) and these values did not decrease further after the fumigation (**Figure 2A**). Ozone also induced a strong decrease in *g*_s_ in WW+O_3_ and even more in WS+O_3_ leaves at the end of the exposure (-13 and -38% in WW+O_3_ and WS+O_3_ leaves, respectively) and the effect of O_3_ on *g*_s_ remained at 24 and 48 h FBE (**Figure 2B**). However, in WS+O_3_ plants, *g*_s_ values were 50% lower than those recorded in WW+O_3_ plants. Finally, C_i_ values increased significantly following O_3_ exposure in both WW+O_3_ and WS+O_3_ leaves (+8%) although the values recorded in WS+O_3_ leaves were significantly higher compared to those found in WW+O_3_. In both WW+O_3_ and WS+O_3_ leaves, the *C*_i_ values reached at the end of the exposure were maintained up to the end of the experimental period, although a slight decrease was observed for WW+O_3_ leaves at 24 h FBE (**Figure 2C**).

Actual Φ_PSII_ decreased at the end of exposure in both WW+O_3_ and WS+O_3_ leaves (-19 and -62%, respectively). However, Φ_PSII_ recovered completely 48 h FBE in both WW+O_3_ and WS+O_3_ leaves (**Figure 2D**). In WW+O_3_ plants, qP values were higher than those observed before the beginning of exposure, both at 24 and 48 h FBE (+19 and +14%, respectively) (**Figure 2E**). Conversely, in WS+O_3_ plants, qP values decreased at the end of exposure (-47%), but recovered completely from 24 h onward (**Figure 2E**). Values of qNP increased in WW+O_3_ leaves at the end of the fumigation (+24%), and similar values were maintained until 48 h FBE (**Figure 2F**). Conversely, in WS+O_3_ plants, qNP decreased at 24 and 48 h FBE (–14 and –8%, respectively, in comparison with the pre-treatment values) (**Figure 2F**). These mechanisms were sufficient to protect PSII from photoinhibition given that the decrease in *F*_v_/*F*_m_ observed at the end of exposure in both WW+O_3_ and WS+O_3_ leaves was completely recovered 48 h FBE (data not shown).

#### Ozone-Induced ROS Accumulation

**FIGURE 3:**
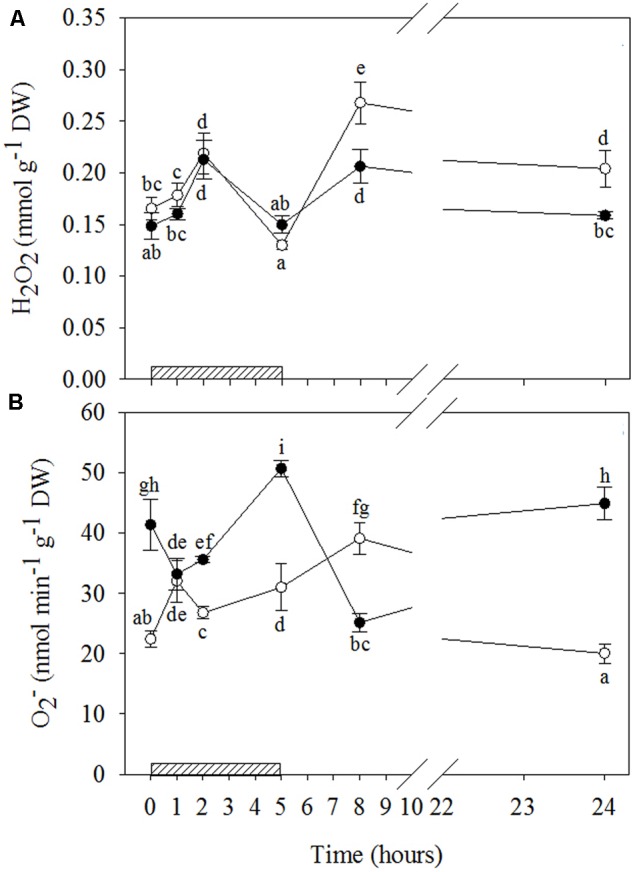
Time course of reactive oxygen species (ROS) in *Quercus ilex* plants well-watered (open circle) or water stressed (20% of the effective evapotranspiration daily for 15 days, closed circle) and exposed to acute ozone (200 nL L^-1^ for 5 h). Data are shown as mean ± standard deviation. The measurements were carried out at 0, 1, 2, 5, 8, and 24 h from the beginning of exposure. According to the two-way ANOVA with treatment and time as variability factors, different letters indicate significant differences (*P* = 0.05). DW, dry weight; H_2_O_2_, hydrogen peroxide **(A)**; $O_{2}^{–}$, superoxide anion **(B)**. The thick bottom line indicates the time (5 h) of ozone exposure.

A biphasic time course of H_2_O_2_ production in response to O_3_ was observed irrespective of drought stress (**Figures 3A** and Supplementary Table S2). In both WW+O_3_ and WS+O_3_ plants, H_2_O_2_ content increased at 2 h FBE (+33 and +43% as compared to time 0, respectively), showed a significant decline at 5 h FBE, and increased again at 8 h FBE. This second peak was higher in WW+O_3_ than in WS+O_3_ plants (+62% vs. +38%, compared to their respective time 0). In addition, only in WW+O_3_ leaves was the second peak higher than the first, and H_2_O_2_ levels at 24 h FBE remained higher than those at time 0 (+24%).

The time patterns of $O_{2}^{–}$ induced by O_3_ were notably different in relation to water stress (**Figures 3B** and Supplementary Table S2). In WW+O_3_ conditions, $O_{2}^{–}$ content also exhibited a clear biphasic time course. It peaked already at 1 h FBE (+47%, compared to the beginning of O_3_-exposure), and again at 8 h FBE (+75%), although it remained higher than time 0 at 2 and 5 h FBE (+20 and +38%, respectively). At 24 h FBE, the $O_{2}^{–}$ content decreased at the same levels as time 0. Conversely, in WS+O_3_ plants (where $O_{2}^{–}$ levels were already higher in WS+O_3_ than WW+O_3_ plants), $O_{2}^{–}$ content decreased during the first two hours of O_3_ treatment (–20 and –14%, after 1 and 2 h FBE, respectively). It then peaked at 5 h FBE (+22%), decreased again at 8 h FBE (reaching the lowest values of the whole treatment), and finally increased again reaching the levels shown at time 0.

#### Ozone-Induced Signaling Molecule Stimulation

**FIGURE 4:**
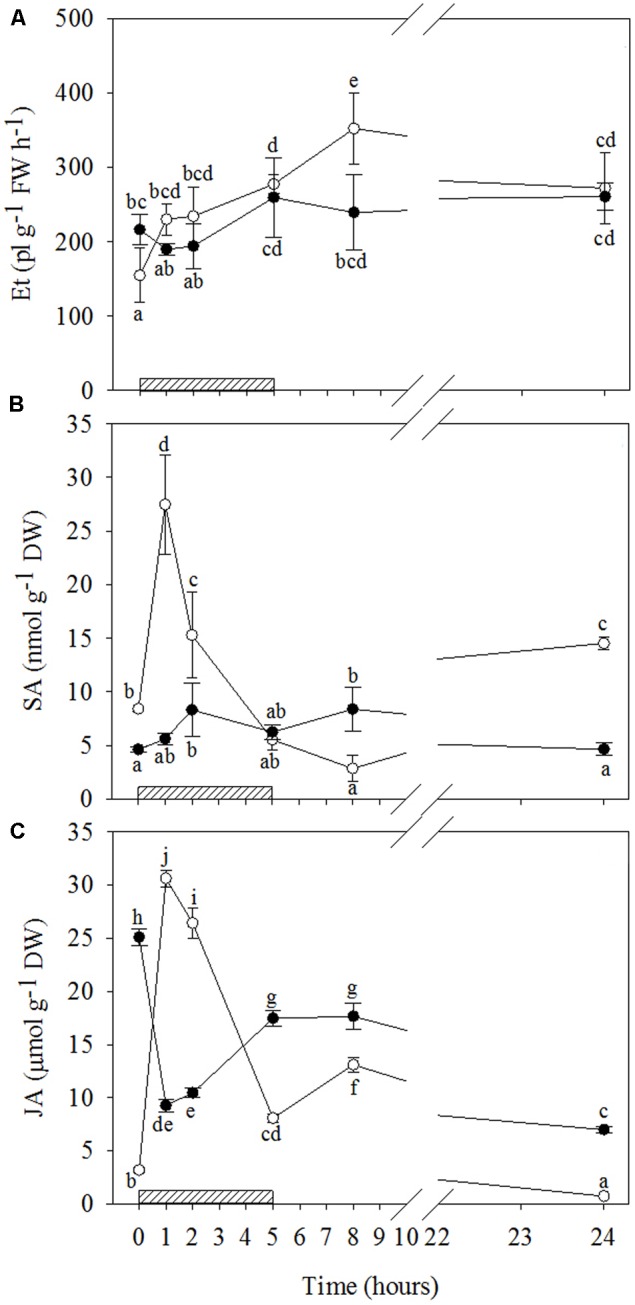
Time course of phytohormones in *Quercus ilex* plants well-watered (open circle) or water stressed (20% of the effective evapotranspiration daily for 15 days, closed circle) and exposed to acute ozone (200 nL L^-1^ for 5 h). Data are shown as mean ± standard deviation. The measurements were carried out 0, 1, 2, 5, 8, and 24 h from the beginning of exposure. According to the two-way ANOVA with treatment and time as variability factors, different letters indicate significant differences (*P* = 0.05). DW, dry weight; ET, ethylene **(A)**; FW, fresh weight; JA, jasmonic acid **(C)**; SA, salycilic acid **(B)**. The thick bottom line indicates the time (5 h) of ozone exposure.

The results of signaling molecules indicate that O_3_ only significantly stimulated ET emission in WW+O_3_ leaves (**Figures 4A** and Supplementary Table S2). In these plants, starting from 1 h of treatment onwards, ET emission values remained higher than those shown at the beginning of O_3_-exposure throughout the treatment period, and reached the maximum 8 h FBE (+49, +52, +79, +128, and +76% after 1, 2, 5, 8, and 24 h FBE, respectively). Conversely, a clear biphasic time course of SA production was observed in response to O_3_, irrespective of drought stress. However, the average concentration throughout the treatment and the changes induced by O_3_ were higher and more pronounced in WW+O_3_ than in WS+O_3_ plants, respectively (**Figures 4B** and Supplementary Table S2). In WW plants, total SA levels increased already at 1 h FBE, reaching their maximum values (three-fold higher than before the O_3_-treatment). They then progressively decreased to constitutive levels at 5 h FBE, and to even lower values at 8 h FBE, but increased again at the end of the experiment (+73% compared to time 0). In WS+O_3_ conditions, SA concentrations peaked at 2 and 8 h FBE (+79 and +80%, respectively), whereas SA levels were similar before the beginning of O_3_ treatment than at the other analysis times.

The time patterns of JA induced by O_3_ were also notably different in relation to drought (**Figures 4C** and Supplementary Table S2). A biphasic time course of JA production was shown by WW+O_3_ plants. Similarly to SA (and ABA, as reported below), a first marked peak in JA levels (tenfold higher than controls) was shown by WW+O_3_ plants at 1 h FBE. Then, JA progressively decreased until 5 h FBE (remaining at higher levels than those recorded at time 0), peaked again 8 h FBE (four times higher than time 0), and, finally, reached lower values than before the beginning of O_3_-treatment at 24 h FBE. Conversely, in WS+O_3_ plants (where JA levels, similarly to $O_{2}^{–}$, were already higher in WS+O_3_ than WW+O_3_ plants) a marked decrease in JA concentrations was observed starting from 1 h onwards (–63%, in comparison to controls). Throughout the period of O_3_-treatment, the values of this phytohormone remained lower than those shown before the exposure, although a recovery was shown at 5 and 8 h FBE.

#### Ozone-Induced ABA and Osmolyte Accumulation

**FIGURE 5:**
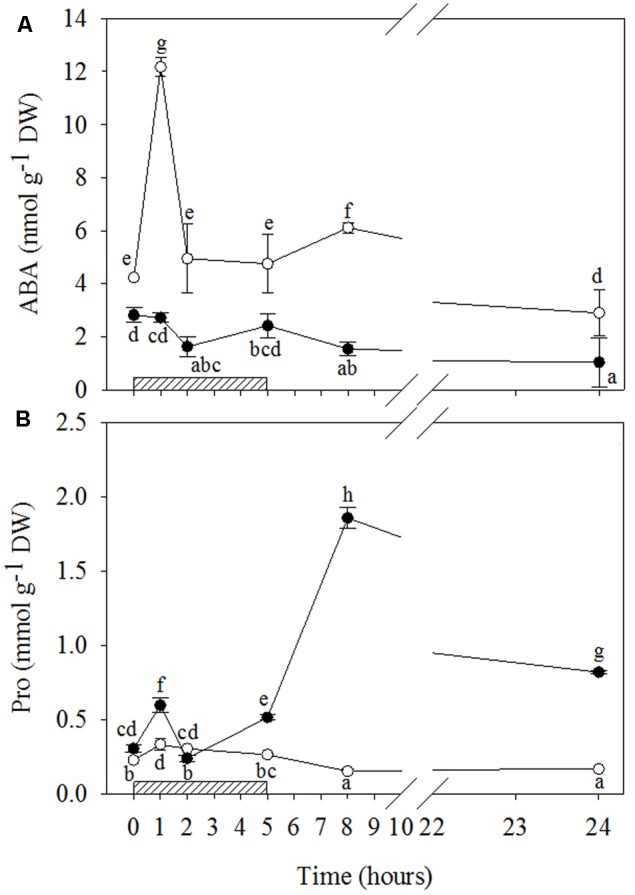
Time course of abscisic acid (ABA; **A**) and proline (Pro; **B**) in *Quercus ilex* plants well-watered (open circle) or water stressed (20% of the effective evapotranspiration daily for 15 days, closed circle) and exposed to acute ozone (200 nL L^-1^ for 5 h). Data are shown as mean ± standard deviation. The measurements were carried out at 0, 1, 2, 5, 8, and 24 h from the beginning of exposure. According to the two-way ANOVA with treatment and time as variability factors, different letters indicate significant differences (*P* = 0.05). DW, dry weight. The thick bottom-line indicates the time (5 h) of ozone exposure.

O_3_ significantly stimulated ABA production only in WW+O_3_ leaves (**Figures 5A** and Supplementary Table S2), where a clear biphasic response to the pollutant was observed. In comparison to the levels shown before the O_3_ treatment, ABA in WW+O_3_ leaves already increased at 1 h FBE (overall three times), showed no differences at 2 and 5 h FBE, slightly increased again at 8 h FBE (+44%) and, finally, reached lower values at 24 h FBE. In WW+O_3_ conditions, O_3_ induced a slight increase in Pro only at the first two hours of exposure (+46 and +33%, respectively at 1 and 2 h FBE), whereas during the post-fumigation period, Pro values remained lower than those at time 0 (**Figures 5B** and Supplementary Table S2). Conversely, in WS+O_3_ plants, Pro content peaked after 1 h FBE (+96%, in comparison to controls), then declined at 2 h FBE (at the same concentrations shown before the beginning of O_3_-exposure), increased again at 5 h FBE (+69%) and reaching a maximum at 8 h FBE, with the maximum values (sixfold higher than at time 0). Finally, Pro concentration of WS+O_3_ leaves decreased at 24 h FBE, although they remained higher than before the O_3_-exposure (more than two fold).

## Discussion

In this study, the behavior of the Mediterranean evergreen *Q. ilex* subjected or not to drought, and later exposed to an acute O_3_ exposure, was evaluated in terms of cross-talk responses among signaling molecules. The aim was to confirm or disentangle the hypothesis according to which drought stress influences the responses of plants to acute episodes of O_3_ exposure.

### Physiological and Biochemical Responses to Drought

Although drought induced a strong decrease in PDΨ_W_ (reaching lower values than those reported in a previous study; Cotrozzi et al. (2016b), attributable to different growing seasons between experiments), the RWC of WS leaves did not significantly change in comparison to the WW leaves. This indicates that a good level of leaf hydration was also maintained under drought conditions, RWC being a reliable indicator of leaf water content (Rosales-Serna et al., 2004). This result is in accordance with the accumulation of Pro, a metabolite that is considered an important compatible solute which (i) facilitates water absorption by increasing the cell osmotic potential (Ashraf and Foolad, 2007), and (ii) reduces cell damage (Filippou et al., 2014). The important role of Pro in response to water stress has already been reported in this species where Pro played a key role in the high plasticity of *Q. ilex* under a long period of moderate water stress (Cotrozzi et al., 2016b).

In WS plants, the strong decline in CO_2_ photo-assimilation was attributable to coordinated and concomitant stomatal and mesophyll limitations, which is in line the results obtained by several authors (e.g., Centritto et al., 2009; Flexas et al., 2013). The drop in A levels was higher than that in *g*_s_, thus leading to lower values of intrinsic water use efficiency (data not shown), as already reported in this species when subjected to water stress (Cotrozzi et al., 2016b).

These outcomes indicate that CO_2_ assimilation was strongly influenced not only by stomatal behavior but also by mesophyll limitations, as demonstrated by the increase in C_i_. Drought compromised the PSII photochemical efficiency in light adapted leaves with decreases in Φ_PSII_ and qP levels, although this inhibition did not determine PSII photoinhibition, as confirmed by unchanged values in the F_v_/F_m_ ratio. In WS conditions, the lower CO_2_ assimilation rate induced, in turn, a reduced consumption of ATP and NADPH synthesized into the chloroplasts and, consequently, led to an excess of excitation energy in the thylakoid membrane, which was only partially dissipated, via non-photochemical mechanisms (increase in qNP). The remaining excess of reducing power in WS plants altered the ROS levels (although H_2_O_2_ did not change, a strong increase of $O_{2}^{–}$ was observed). This led to a modification in phytohormones and other signaling molecule cross-talk in terms of (i) promoting oxidative stress and (ii) modulating leaf senescence (Munné-Bosch and Alegre, 2004; Miller et al., 2010; Baxter et al., 2014), which is a defense commonly activated in response to both a plethora of abiotic and biotic stresses (Wingler and Roitsch, 2008).

Among phytohormones, the important roles of ABA and ET in plant responses to drought is well known (Munné-Bosch and Alegre, 2004) as ABA represents the most important regulator of stomata functioning (Wilkinson and Davies, 2002), whereas ET is a shoot growth inhibitor and a promoter of ripening, senescence and abscission (Abeles et al., 1992). However, ET can inhibit ABA-induced stomatal closure (Tanaka et al., 2005). Wilkinson and Davies (2009, 2010) reported that under stress-oxidative conditions, an ET-antagonism of the stomatal response to ABA occurs. This interaction was confirmed by our data (ABA decreased, while ET increased), suggesting that drought-induced ET biosynthesis could be considered a marker of leaf senescence (Dangl et al., 2000; Dolferus, 2014).

In addition, SA and JA have been shown to play a central role in leaf senescence (Abreu and Munné-Bosch, 2008), although they are well known for triggering defense reactions against biotrophic and necrotrophic pathogens such as induced resistance (Barna et al., 2012; Shigenaga and Argueso, 2016). In particular, SA and JA interact at physiological levels in many growth and developmental processes, and they play a role in controlling gene expression during leaf senescence (Abreu and Munné-Bosch, 2008). However, as only the JA levels increased in WS leaves, it is reasonable that only JA participated in senescence-associated signaling and degradative processes of membranes. The significantly higher levels of JA shown by WS compared to WW plants indicate that lipid peroxidation producing substrates for octadecanoid pathways was exacerbated in limited water conditions. In particular, JA could be a promoter of leaf senescence in response to drought, thus leading to stomatal closure and accumulation of osmo-compatible solutes (in our case, only Pro), in line with Dar et al. (2015).

### Influence of Drought Stress on the Physiological and Biochemical Responses to O_3_ Exposure

The physiological responses observed in O_3_-stressed plants were similar to those shown at the end of water deprivation, and in accordance with a previous study by our research group on *Q. ilex* exposed to O_3_ (Cotrozzi et al., 2016b). At the end of the fumigation, the O_3_-induced stomatal closure found in both WW+O_3_ and WS+O_3_ leaves led to significant reductions in CO_2_ assimilation. The more pronounced decrease in A in WW+O_3_ compared to WS+O_3_ leaves, as well as the lack of a further decrease in A observed in WS+O_3_ plants throughout the recovery phase, was probably attributable to the very low CO_2_ assimilation rate shown by water stressed plants before the beginning of the fumigation. The increase in C_i_ level in plants exposed to O_3_ indicates that the pollutant gas influenced not only stomatal conductance but, as with after water stress, also the mesophyll activity. In fact, an impairment of PSII activity was recorded. Although *F*_v_/*F*_m_ and Φ_PSII_ values decreased significantly after O_3_ exposure in both WW+O_3_ and WS+O_3_ plants, the reduction was more pronounced in WS+O_3_ plants. This behavior was linked to different quenching responses to the leaf-water status of plants. In WW+O_3_ plants, where qP did not decrease, a mechanism aimed at dissipating the excess excitation energy was activated (qNP increased). By contrast, in WS+O_3_ leaves, the O_3_-induced decrease in qP was ascribable to the fact that qNP values were already high (probably at their maximum in relation to the capability of plants to activate this mechanism) after drought, and the leaves were not able to enhance this type of dissipation mechanism. The complete recovery of PSII photochemical activity during the recovery time after drought and O_3_ exposure indicates that the decrease in PSII activity was sufficient to prevent the photosynthetic apparatus from undergoing irreversible damage.

Unlike the ecophysiological measurements, microscopic analyses highlighted significant differences between plants exposed (WW+O_3_, WS+O_3_) or not (WW and WS) to the gaseous pollutant. Although visible symptoms were not shown by any of the plants irrespectively of the applied treatment, DAB staining and Evan’s blue incorporation observed in WW+O_3_ and WS+O_3_ leaves 5 h FBE indicated that H_2_O_2_ deposition and cell death had already occurred at the end of exposure. This confirms that O_3_ resembles the HR occurring in incompatible plant-pathogen interactions (Iriti and Faoro, 2008; Vainonen and Kangasjärvi, 2015). An integrated perspective has been proposed to explain how phytohormones and signaling molecules might be involved in molecular events (namely lesion initiation, propagation, and containment) leading to O_3_-induced HR-mimicking foliar symptoms (Overmeyer et al., 2003, 2005; Kangasjärvi et al., 2005). ROS, phytohormones and other signaling molecules have a pivotal role in both HR-mimicking responses induced by acute O_3_ and in promoting leaf senescence under drought (as shown by WS plants). The trends of these molecules were monitored in both well-watered and drought-stressed plants during and after O_3_ exposure, in order to test the hypotheses of this work.

Given that O_3_ induces an endogenous, active and self-propagating ROS generation in the apoplast and a subsequent cellular oxidative burst, some authors have proposed that short-term O_3_ exposure mimics pathogen infection (Rao et al., 2000; Kangasjärvi et al., 2005; Carmody et al., 2016). The two O_3_-induced H_2_O_2_ peaks observed in our saplings, irrespectively of the water conditions, are analogous to the biphasic response usually observed during the establishment of the HR of plants against pathogens. The first H_2_O_2_ peak usually reflects elicitation by pathogen-associated molecular patterns, and the second reflects the interaction between a pathogen-encoded virulence gene product with a plant resistance gene (Mur et al., 2009). In our study, the first peak observed during the fumigation was attributable to O_3_-decomposition, whereas the second peak, in the recovery period, could be entirely ascribable to the plant metabolism, in line with Mahalingam et al. (2006), Di Baccio et al. (2012), and Pellegrini et al. (2013) in herbaceous species.

Although the similarity in H_2_O_2_ profiles over time between WW+O_3_ and WS+O_3_ conditions, the divergence in the magnitude of their relative peaks (the second peak of the WW+O_3_ plants was much greater than their first peak and greater than the second peak of the WS+O_3_ plants, where the two peaks were not significantly different from each other) suggests that drought partially inhibited the response to O_3_-stress. As H_2_O_2_ is one of the most important products of oxidative stress (Gill and Tuteja, 2010), it is reasonable to speculate that the biphasic trend of H_2_O_2_ observed in *Q. ilex*, irrespectively of drought stress, might reflect the biphasic oxidative burst in response to O_3_, in line with several authors (e.g., Wohlgemuth et al., 2002; Di Baccio et al., 2012).

However, this hypothesis is strengthened by $O_{2}^{–}$ only in WW+O_3_ plants, where a biphasic time course of $O_{2}^{–}$ levels was shown concurrently with H_2_O_2_. Kangasjärvi et al. (2005) reported similar temporal changes in ROS for O_3_-sensitive genotypes of several species (e.g., tobacco, tomato, birch), whereas only a modest increase in the first hours of exposure was observed in O_3_-tolerant genotypes. Conversely, the different $O_{2}^{–}$ patterns of the WS+O_3_ plants suggest a possible dual function of this radical depending on water stress. In fact, the significant decrease in $O_{2}^{–}$ observed in WS+O_3_ plants during the first hours of exposure suggests that under drought+O_3_ superoxide anion may act as a precursor of H_2_O_2_ and even more toxic radical derivatives. However, the accumulation of $O_{2}^{–}$ had already been induced by drought before the beginning of O_3_-exposure. On the other hand, the marked increase in $O_{2}^{–}$ content at the end of the exposure demonstrates that this radical may also be directly involved in the O_3_-oxidative burst.

Reactive oxygen species should not be considered as exclusively deleterious and harmful. They can (i) play a key role in intracellular communication which triggers the acclimation ability, and (ii) indirectly orchestrate PCD (Mittler et al., 2011; Xia et al., 2015; Carmody et al., 2016). The amplification of ROS signals and the complete induction of defense genes seem to require signal molecules (Overmeyer et al., 2003). The differences observed in the present study in O_3_-induced ROS extent dynamics in relation to water stress suggest a rather complex network of events in signal transduction, involving other molecules (e.g., phytohormones) and processes. Metabolites such as ET, ABA, SA and JA may interact at the physiological level in many growth and developmental processes, with a key role in controlling gene expression during leaf senescence. Most of the genes regulated by these metabolites are defense-related (Fossdal et al., 2007), participating therefore in responses to O_3_ (Xu et al., 2015).

Under both biotic and abiotic stresses, SA is required for the induction of PCD, controlling and potentiating the oxidative burst together with ET, whereas JA is involved in limiting the spread of lesions (van Loon et al., 2006; Shigenaga and Argueso, 2016). There are three phases that highlight the influence of ABA on stress conditions (Rejeb I.B. et al., 2014). First, ABA induces stomatal closure, which leads to a reduction in water loss (in this phase, SA, JA and ET may not yet be activated and ABA can antagonize their induction). In the second step, there is a post-infection reaction- an intact ABA signaling pathway is required to increase callose accumulation in affected plants, and the presence of ABA can induce or repress additional callose accumulation depending on the environmental conditions. The third phase begins when pathogen-associated molecular patterns stimulate the accumulation of specific SA, JA, and ET hormones in order to regulate the defense reaction.

In our study, the patterns of phytohormones during and after O_3_ treatment were completely different in WW+O_3_ and WS+O_3_ plants, showing how drought stress has a pivotal role in O_3_ responses, and how these signal molecules may be altered in relation to water stress. The ET and ABA accumulations observed throughout the entire period of O_3_ exposure occurred only in well-watered conditions. On the other hand, when plants had been previously subjected to water stress, their unchanged values suggest that ET and ABA were not involved in either signaling-responses to O_3_, or senescence strategies (as shown for WS plants).

It is worth noting that (i) the maximal ET emission in WW+O_3_ plants coincided with the second peak of H_2_O_2_ and $O_{2}^{–}$; (ii) the first peak of ABA (during O_3_ treatment) preceded that of H_2_O_2_, suggesting that ABA could act as a stress messenger by inducing H_2_O_2_ (Jiang and Zhang, 2002) and consequently stomatal closure (as confirmed by the decrease in g_s_ values observed at the end of exposure), and (iii) the weaker second ABA peak (in the recovery phase) was concomitant with the maximum H_2_O_2_ and $O_{2}^{–}$ levels and the maximal ET emission. These outcomes confirm a spatial and functional correlation between ROS and the accumulation of these phytohormones.

The SA induction observed, irrespectively of whether the plants had been subjected to drought or not, suggests that this metabolite is also an important modulator of O_3_-induced responses (Pasqualini et al., 2002; Horváth et al., 2007). However, the differences between WW+O_3_ and WS+O_3_ plants show that the functioning of SA is dependent on water stress. In WW+O_3_ conditions, the strong increase in SA during the first hours of the treatment and at the end of the O_3_ fumigation, confirms the central role of this metabolite in lesion initiation and progression in response to O_3_ (Tamaoki, 2008). In addition, the greater SA concentration corresponded with the maximal ABA stimulation and the first increase in ET, thus demonstrating the synergistic action of these hormones in the regulation of defense reactions (Roychoudhury et al., 2013; Wang et al., 2013). By contrast, the biphasic time course of SA (similar to that of H_2_O_2_) shown by WS+O_3_ plants (although slight) recalls the biphasic induction that develops during biotrophic pathogen infection (Mur et al., 2009). Here, the similarity in magnitude of the two SA peaks suggests that the first accumulation of this metabolite (concomitant with the first peak of H_2_O_2_) did not actuate the second increase in H_2_O_2_ and hence did not affect the level of plant defense.

The highest concentration of JA observed in WW+O_3_ plants during the first hour of fumigation coincided with the initial increase in ET and the maximum accumulation of SA and ABA, thus also demonstrating a spatial and functional correlation between these compounds (Thaler et al., 2012). The significant O_3_-induced decrease in JA levels observed in WS+O_3_ plants during and after the exposure suggests that JA did not promote leaf senescence in O_3_-treated leaves in spite of the high concentrations of this metabolite observed in WS+O_3_ plants, not excluding the involvement of JA in senescence-associated signaling (Abreu and Munné-Bosch, 2008). In fact, the JA level in WS+O_3_ plants increased again after the end of fumigation, reaching higher values than those found in the WW+O_3_ counterpart during the recovery. JA is known to rapidly inhibit the expression of genes involved in photosynthesis by inducing chlorophyll loss and cellular changes that cause less photochemical damage (Santino et al., 2013).

Proline plays several roles in plant responses to abiotic and biotic stresses, and under stress its metabolism is affected by multiple and complex regulatory pathways which can profoundly influence cell death and survival in plants (Zhang and Becker, 2015). The slight increase in Pro observed in WS plants compared to WW likely indicates its role as an osmoprotectant. By the same token the O_3_-induced increase in Pro observed in WW+O_3_ plants only during the first hours of treatment and coinciding with the maximal ABA, SA and JA stimulation and the first increase in ET, H_2_O_2_ and $O_{2}^{–}$, suggests a potential cross-talk among signaling molecules in regulating Pro metabolism, as previously reported by Rejeb K.B et al. (2014). The role of Pro as an ROS scavenger has also been reported (Szabados and Savouré, 2009; Banu et al., 2010; Zhang and Becker, 2015). In WW+O_3_ plants the lack of accumulation in Pro at 8 h FBE was concurrent with a strong increase in H_2_O_2_, whereas in WS+O_3_ the huge increase in Pro at 8 h FBE suppressed the increase in H_2_O_2_ (which remained at the same levels shown at 1 h FBE), thus suggesting an H_2_O_2_-scavenging role in these water conditions. This mechanism was also confirmed at 24 h FBE. Several studies have attributed an antioxidant feature to Pro, suggesting the capability of Pro in $O_{2}^{–}$ and H_2_O_2_ quenching (e.g., Szabados and Savouré, 2009; Wang et al., 2009).

## Author Contributions

The work presented here was carried out in collaboration among all authors. GL and RM defined the research theme and obtained funding. LC, DR, EP, MT, AT, and ML designed methods, carried out laboratory experiments and analyzed the data. LG, CN, and PV co-designed experiments, discussed analyses, interpreted the results, and wrote the paper. All authors have contributed to, seen and approved the manuscript.

## Conflict of Interest Statement

The authors declare that the research was conducted in the absence of any commercial or financial relationships that could be construed as a potential conflict of interest.
