# Supplementary material for: Losing the Warning Signal: Drought Compromises the Cross-Talk of Signaling Molecules in Quercus ilex Exposed to Ozone
Source: Front Plant Sci. 2017 Jun 15;8:1020. doi: 10.3389/fpls.2017.01020 (PMC5475409; doi:10.3389/fpls.2017.01020)
Supplement: Supplementary file 1 [file Table_1.DOCX]

**Table S1.** *F* values of one-way repeated measures ANOVA of the effects of acute ozone exposure (200 nL L^-1^ for 5 h) in time (0, 5, 24 and 48 h from the beginning of the exposure) on CO_2_ assimilation rate (A), stomatal conductance to water vapor (g_s_), intercellular CO_2_ concentration (C_i_), PSII photochemical efficiency in dark (F_v_/F_m_) andlight conditions (Φ_PSII_), photochemical quenching (qP) and non-photochemical quenching (qNP) coefficients in Quercus ilex plants well-watered or water stressed (20% of the effective evapotranspiration daily for 15 days). Asterisks show the significance of factors/interaction: *** P ≤ 0.001, ** P ≤ 0.01, * P ≤ 0.05, ns P > 0.05. d.f. represents the degrees of freedom.

|  | d.f. | A | g_s_ | C_i_ | F_v_/F_m_ | Φ_PSII_ | qP | qNP |
| --- | --- | --- | --- | --- | --- | --- | --- | --- |
| *Treatment* | 1 | 1993.39  *** | 297.48  *** | 804.57  *** | 38.62  ** | 206.87  *** | 96.02  *** | 35.94  ** |
| *Time* | 3 | 101.97  *** | 50.67  *** | 38.14  *** | 9.98  ** | 15.68  *** | 25.90  *** | 5.36  * |
| *Treatment × Time* | 3 | 35.91  *** | 3.00  ns | 6.14  ** | 4.51  * | 0.94  ns | 6.48  ** | 5.32  * |
