# Supplementary material for: Losing the Warning Signal: Drought Compromises the Cross-Talk of Signaling Molecules in Quercus ilex Exposed to Ozone
Source: Front Plant Sci. 2017 Jun 15;8:1020. doi: 10.3389/fpls.2017.01020 (PMC5475409; doi:10.3389/fpls.2017.01020)
Supplement: Supplementary file 2 [file Table_2.DOCX]

**Table S2.** *F* values of one-way repeated measures ANOVA of the effects of acute ozone exposure (200 nL L^-1^ for 5 h) in time (0, 5, 24 and 48 h from the beginning of the exposure) on hydrogen peroxide (H_2_O_2_), superoxide anion (O_2_^-^), ethylene (ET), salicylic (SA), jasmonic (JA) and abscisic (ABA) acid and proline (Pro) in Quercus ilex plants well-watered or water stressed (20% of the effective evapotranspiration daily for 15 days). Asterisks show the significance of factors/interaction: *** P ≤ 0.001, ** P ≤ 0.01, * P ≤ 0.05, ns P > 0.05. d.f. represents the degrees of freedom.

|  | d.f. | H_2_O_2_ | O_2_^-^ | ET | SA | JA | ABA | Pro |
| --- | --- | --- | --- | --- | --- | --- | --- | --- |
| *Treatment* | 1 | 24.98  *** | 134.00  *** | 5.13  * | 77.24  *** | 10.49  ** | 303.37  *** | 2408.73  *** |
| *Time* | 5 | 40.89  *** | 37.13  *** | 8.68  *** | 24.90  *** | 356.00  *** | 46.66  *** | 519.61  *** |
| *Treatment × Time* | 5 | 6.52  *** | 50.37  *** | 3.76  * | 30.67  *** | 736.75  *** | 30.71  *** | 726.21  *** |
